# The Single Midline Implant in the Edentulous Mandible—Current Status of Clinical Trials

**DOI:** 10.3390/jcm12113773

**Published:** 2023-05-31

**Authors:** Nicole Passia, Matthias Kern

**Affiliations:** 1 Department of Prosthodontics, Faculty of Medicine and University Hospital Carl Gustav Carus, Technische Universität Dresden, Fetscherstr. 74, 01307 Dresden, Germany; 2 Department of Prosthodontics, Propaedeutics and Dental Materials, Christian-Albrechts University at Kiel, Arnold-Heller-Str. 3, Haus B, 24105 Kiel, Germany; mkern@proth.uni-kiel.de

**Keywords:** single mandibular implant, edentulous mandible, implant survival rates

## Abstract

The single midline implant in the edentulous mandible is a treatment concept that has often been controversially discussed. Nearly 30 years ago, the first available clinical results revealed high implant survival rates and remarkable improvements in oral comfort, function, patient satisfaction and oral health-related quality of life for edentulous patients compared to the situation with no implant. However, the clinical trials were predominantly conducted with a small number of patients over a short to medium follow-up period. Today, numerous clinical investigations on the single midline implant in the edentulous mandible, which increasingly include longer-term observation periods, are available. It is the aim of this overview to present the current literature and to highlight the clinical problems. This article is a 2023 update of a review published by the authors in the German language in 2021 in the German journal *Implantologie*. In total, 19 prospective clinical trials with a follow-up period of 0.5–10 years were analyzed. Over this observation period, single implants with modern rough implant surfaces in the edentulous mandible reveal high implant survival rates of between 90.9 and 100% when a conventional delayed loading protocol was applied.

## 1. Introduction

According to the Global Burden of Disease study, 267 million people worldwide were edentulous in 2017 [1]. The highest number of prevalent cases was in upper-middle-income countries with 120 million affected people, followed by high-income countries with a prevalence of 74 million people. Edentulism is predominantly present in older adults and is very widespread in industrial countries [2,3,4]. In Europe, the estimated number of edentulous patients in the population aged 65–74 years still varies between 2.7 and 27.6% [5]. Although the number of edentulous patients decreased during the last decades, 12.4% of the younger seniors (65–74 years) and 32.8% of the older seniors (75–100 years) are completely edentulous in Germany [6]. Tooth loss and edentulism are directly related to social and economic status. The lower the social status, the higher the proportion of edentulous seniors [6].

A common treatment option for edentulous jaws is a complete denture. Due to anatomical, physical and other factors, maxillary complete dentures can usually be stabilized adequately. In the mandible, good denture stability is often not achievable due to the frequently advanced alveolar ridge resorption. As a consequence, many edentulous patients complain about the retention and stability of their mandibular dentures, despite a technically adequate design [7,8]. Their chewing ability [9,10] as well as their general and oral health-related quality of life is often severely reduced [11,12]. The chewing ability of a complete denture wearer is between one-third and one-seventh that of the chewing ability of a naturally dentate person [13]. As a result, edentulous patients often switch their dietary intake to softer food [14], which often contains less protein, minerals and vitamins and can lead to malnutrition or undernutrition in the long term [15]. Additionally, social factors have to be considered. Seniors, who are not satisfied with their dentures may withdraw from public, which can result in loneliness or depression [16].

Denture adhesives are able to stabilize a mandibular denture at least up to 4 h after adhesive application [17]. However, a systematic literature review on denture adhesives concluded that adhesives indeed improve the function and stability of complete dentures, but pointed out that long-term studies on the biological effect of denture adhesives are still missing [18]. A clinical investigation on the influence of soft relining overdentures on oral health-related quality of life found a positive impact of the soft relining [19]. However, the possibility of a cytotoxic effect of acrylic-based soft denture relining materials should be considered [20] before recommending their general usage.

The situation of edentulous patients improved significantly with the introduction of dental implants and today two implants in the interforaminal area are considered the standard treatment for the edentulous mandible internationally [21,22]. This treatment option shows very good results with regard to implant survival [23,24], patient satisfaction [25] and masticatory performance [26,27]. Three or more implants in combination with a removable overdenture and four or more implants in combination with a fixed or removable restoration are also well documented in the literature with overall high survival and success rates [23,28,29]. However, these treatment options require long treatment time and a relatively high surgical effort, which must be carefully considered, especially in older patients [30]. In addition, these treatments are associated with considerable costs many cannot afford. These are the main factors that can lead to a negative attitude towards an implant therapy, especially in older seniors [31,32].

To overcome the burden of an invasive surgery and to reduce the treatment costs, the concept of a single implant in the edentulous mandible to support a complete denture was firstly introduced by Cordioli et al. in the 1990s [33]. Since then, it has often been controversially discussed. The first 5-year results from a clinical trial with a small group of edentulous seniors revealed an implant survival rate of 100% [34]. Additionally, a remarkable improvement in the oral comfort and function of the edentulous patients was observed. In the following years, different clinical investigations, predominantly with a relatively small number of patients over a short to medium follow-up period, confirmed high implant survival rates and significant improvements for patient satisfaction, oral health-related quality of life and masticatory performance compared to the situation without an implant [35,36,37,38,39]. 

Additionally, different investigations comparing one implant with two or more implants have been conducted [40,41,42,43]. Walton et al. provided edentulous patients with either one or two implants in the edentulous mandible [44]. They found no statistically significant differences in patient satisfaction or follow-up interventions between the two treatment groups. Material costs and treatment time were lower in the group that received one implant. In another clinical trial comparing one central implant and two implants in the interforaminal area, patient satisfaction and mean marginal bone loss in the area of the implants were investigated [40]. After 12 months, patient satisfaction was high with no statistically significant difference between the two groups and the mean marginal bone loss was also comparable for both groups. 

However, the concept of a single implant still does not seem to be widely used. According to an international survey in 2019 among prosthodontists from the International College of Prosthodontists involving 116 dentists worldwide regarding the use of mandibular implant-supported prostheses, only one respondent used a single implant [45].

To date, numerous clinical investigations on the single midline implant have been published, which increasingly include longer-term observation periods of five and more years [46]. Therefore, the aim of this overview is to present the current literature on the single implant in the edentulous mandible and to highlight the clinical problems.

## 2. Current State of Investigations

Based on a systematic review in 2021 [46], we conducted a search of the Medline international database (www.pubmed.com, accessed on 14 April 2023) Web of Science and Scopus (both 26 May 2023) for publications on the single midline implant in the edentulous mandible published between January 2021 and April 2023 using the following search terms: ‘single’ AND ‘midline’ AND ‘implant’ AND ‘mandibular’ AND ‘denture’, ‘single’ AND ‘dental’ AND ‘implant’ AND ‘mandibular’ AND ‘denture’, ‘single’ AND ‘median’ AND ‘implant’ AND ‘mandibular’ AND ‘denture’ in multiple variations. Prospective clinical trials published in English with a follow-up period of at least 6 months were included. Case reports, review articles and laboratory investigations on this treatment option were not taken into account. Two new reviews [39,47], two study updates [43,48] and four new clinical trials [49,50,51,52] were found. Thirteen of the clinical trials published before 2021 [34,37,38,40,42,53,54,55,56,57,58,59,60] did not have further published follow-ups (Table 1). A total of 19 investigations, with a mean follow-up period between 6 months and 9 years reporting on 547 patients were included. The majority of these received a ball attachment (439 patients); in 67 cases, a Locator stud-attachment (Locator, Zest Anchors, Escondido, CA, USA) was used; 32 patients were treated using a stud-attachment; and another 5 patients each received a magnet attachment or a Novaloc attachment.

In seven investigations including 110 implants, a conventional loading protocol with implant loading between 3 and 5 months after implant insertion was chosen [34,38,49,50,52,54,55]. All implants survived over a mean observation period between 1 and 5 years. In one investigation with a conventional loading protocol comparing one versus two implants, one implant failed in each group [57]. In the single implant group with 11 patients, one implant had to be removed during second-stage surgery before implant loading; the implant from the two-implant group with 10 patients failed 3 months after the surgery, resulting in a survival rate of 90.9% for the one-implant group and 95% for the two-implant group, respectively. In another clinical trial with 22 patients, a conventional loading protocol was chosen [59]. One implant failed after two months, resulting in an implant failure rate of 4.5% after 12 months of observation.

In three other investigations, implants were loaded immediately after insertion [37,48,56]. The implant survival rate was 82.4–100% after 3–5 years of observation when implants with rough surfaces were used. In one of the aforementioned trials, eight implants with machined surfaces were inserted and immediately loaded. After three years, 37.5% of the implants had failed, all within the first 8 weeks after implant placement [61].

In a multicenter clinical trial including 158 patients, 81 implants were loaded conventionally three months after implant placement, and 77 implants were loaded immediately at the day of implant surgery [60]. After 5 years of observation, 9 implants had failed in the immediate loading group, all within the first three months after implant loading, and two implants had failed in the conventional loading group, resulting in an overall survival rate of 87.8% for the immediate loading group and 97% for the conventional loading group. 

In five investigations, an open healing protocol was chosen and implants were loaded moderately over the healing abutment [40,42,43,51,53]. After 3–6 weeks, the retention elements were inserted. Four of the aforementioned investigations including 43 patients reported an implant survival rate of 100% after a mean observation period of 0.5–5 years [40,42,43,51]. In one investigation including 36 patients, implants from three different systems were inserted. After 12 months, the implant survival rate was 91.7% and all implant failures occurred with one specific implant system [53].

In a clinical trial with 11 patients, six implants were conventionally loaded after three months, and 5 implants were moderately loaded over a long healing abutment at the day of implant insertion [58]. Over a mean observation period of 9 years, no implant was lost.

The loading protocol of the single mandibular implant does not seem to have an influence on prosthodontic maintenance interventions. The most common procedures in the available clinical trials were activation or replacement of the retention element as well as relining of the denture base [42,58,60]. No significant differences in the incidence of prosthodontic events were found between dentures retained by one or two implants [42,43].

A direct comparison of single-implant overdentures, two-implant overdentures and four-implant fixed dental prostheses found higher rates of maintenance visits for the overdentures with matrix replacements as the most common maintenance intervention [49].

Another frequent complication was a fracture of the denture base, predominantly in the area of the retention element, in prostheses without metal framework reinforcement. While some investigations found fractures in up to 36% of the cases [42,48,53,58,62], other investigations did not report on any fracture of the denture base [34,38]. In a retrospective investigation on overdentures retained by either one or two implants for at least 17 months, the prosthesis fracture rate with one implant was with 21.4% more than twice as high as for two implants with 9.2% [41]. A similar result was reported from a prospective clinical trial with a fracture rate of 41% for one implant and 21% for two implants after 5 years of observation [42]. Thus, an increased risk of fracture can be assumed for one implant compared to two implants in prostheses without metal framework reinforcement.

Investigations reporting on oral health-related quality of life or patient satisfaction found an improvement after implant placement compared to the initial situation with no implant [34,37,38,42,57,63]. While some investigations found no differences in patient satisfaction for overdentures retained by one or two implants [40,42,64], others found higher satisfaction scores for two implants than for one implant [57,65].

The masticatory performance of edentulous patients significantly improved after implant insertion compared to the situation with sufficient complete dentures [50,57,64,66,67,68,69]. Masticatory performance was measured using different methods such as chewing of almonds, carrots or an artificial test food followed by sieving of the chewed food with sieves of different sizes, or measuring the mixing ability of a two-colored chewing gum. Direct comparison of therapy with 1 or 2 implants showed greater improvement in masticatory function with the use of two implants in two investigations [50,57], while another clinical trial found no inferiority with the single implant therapy [64].

In another clinical trial, the influence of the number of implants on chewing efficacy was investigated [50]. Thirteen patients received three implants in the edentulous mandible, which were successively loaded. Chewing efficacy clearly improved after loading the first implant. It slightly further improved after loading the second implant.

## 3. Discussion

The available clinical trials on the single mandibular implant show very high implant survival rates over a medium- to long-term period of up to 10 years, provided that rough implant surfaces are used and immediate implant loading via the retention element is avoided. An open healing and a moderate loading of the implant over the healing abutment does not seem to negatively influence implant survival. However, the relatively small number of patients in most of the investigations has to be considered. In randomized clinical trials comparing 1 or 2 implants to support an overdenture, there were also no significant differences between the treatment groups with regard to implant survival rates or prosthodontic events, especially with regard to the maintenance of the retention element [40,42,43,49,56,57].

A direct comparison of overdentures retained with 1 or 2 implants and fixed dental prostheses retained with 4 implants clearly revealed the retention element as the “weak” part of the overdenture, which requires frequent maintenance care [49]. However, the retention element can be considered a wear part, which requires regular maintenance and occasional replacement. According to different literature reviews, this is the most common maintenance intervention for any implant overdenture treatment [70,71].

The area of the retention element must also be considered the weak link with regard to fractures of the denture base, which predominantly occurred in the implant area of dentures without metal framework reinforcement [37,41,46,58]. In most investigations, the retention element was incorporated into the existing prostheses or the prostheses newly fabricated as part of the study, which were not reinforced with a metal framework. Under masticatory loading, the prosthesis fractures in the most fragile area, the region of the implant, which is further weakened by the incorporation of the matrix. If single-implant overdentures fracture in the anterior area, they should be reinforced with a metal framework, which can easily be performed during the necessary repair process.

Clinical studies investigating the influence of the single midline implant on oral health-related quality of life and satisfaction of edentulous patients came to the unanimous conclusion that the situation clearly improved after loading the implant compared to the situation with no implant [63,66,72,73,74]. For masticatory performance as well as chewing function, similar positive results were detected. A remarkable improvement after implant loading was achieved [66,72,75] and remained stable over years [76]. This seems to be important especially for older seniors, not only to avoid malnutrition or undernutrition with the well-known consequences for general health. Chewing function also seems to have an influence on the intellectual activity as well as the social role of the elderly. Takata et al. investigated the relationship between chewing ability and high-level functional capacity in 80-year-old Japanese seniors [77]. They found a significant correlation between the number of total chewable foods, hard foods or moderately hard foods, and total functional capacity and intellectual activity or social role ability. The authors concluded that maintenance of chewing ability in seniors might result in better intellectual activity and social role. Another recent clinical investigation revealed poor nutritional status and the consumption of soft food as potential risk factors for developing Alzheimer’s disease [78].

A critical aspect of the therapy with the single midline implant in the mandible is the influence of the movement of the implant overdenture and its biomechanical behaviour. In a three-dimensional finite element analysis, the influence of the number of implants on the biomechanical behaviour of mandibular overdentures was analyzed [79]. According to that investigation, single implant overdentures rotate from side to side under vertical loading of the mandibular incisors above the implant. However, no obvious increase in loading in the peri-implant bone was detected. In another laboratory investigation, Oda et al. showed significantly less vertical displacement of a single-implant-supported overdenture compared to a two-implant-supported overdenture under anterior loading [80]. The authors mentioned additionally that the single-implant-supported overdenture can allow complex prosthetic movements under clinical conditions, as a single implant does not limit the direction of the prosthesis movement.

Therefore, patients should be informed before implant placement that a single implant in the edentulous mandible is able to hold the overdenture in place, but that movements of the denture during mastication are still possible, as the implant acts like a potential rotational axis. Patients with a reduced bone height in the posterior region of the mandible in particular might be affected by this. For that reason, patients were excluded from a multicenter clinical trial during screening comparing immediate and conventional loading of a single implant if their bone height in the posterior region of the mandible was less than 11 mm, as it was assumed that those patients would not benefit from a single implant [60]. In the end, nearly 15% of the initially screened 224 patients were excluded due for that reason [81]. However, in another prospective clinical investigation, 18 patients with severely resorbed mandibles received a single short implant. Implants were loaded conventionally three months after implant placement using a stud attachment [52]. Patients’ satisfaction with the overdenture as well as oral health-related quality of life increased significantly after 12 months of observation. However, due to dissatisfaction with the treatment, 11.1% of the patients required additional implants after 12 months to further stabilize the overdenture. They were considered as prosthodontic failures. The authors concluded that further trials with larger patient cohorts and longer follow-up periods were needed to confirm these findings.

The movements of the overdenture over the single implant during mastication might also have an influence on the survival rate of immediately loaded single implants. Implants, which were immediately loaded over the retention element, revealed higher failure rates compared to conventionally loaded implants, and also if the retention element was integrated into the denture base intraorally to avoid a malpositioning of the matrix, which could result in an overload of the implant. In the initial phase after implant insertion, the motion between the implant and the surrounding bone is a high risk factor for early implant loss [80] as at that time the initial mechanical implant stability is gradually replaced by biological stability. Therefore, immediate loading of the implant over the retention element should only be considered in exceptional cases. If a high primary implant stability is achieved and an open healing protocol is chosen to avoid a second-stage surgery, a moderate loading over the healing abutment with a softly relined overdenture does not seem to negatively affect implant survival.

None of the 19 investigations with the single midline implant reported complications with regard to clinically relevant sensory disturbances. According to a cadaver study, it is most likely that the nerve canal running in the center of the anterior mandible, the so called “genial spinal canal”, is hit when a central single implant is inserted [82]. It is possible, however, that the bundle of nerve vessels running in this canal is degeneratively altered to such an extent in edentulous patients [83] that its damage has no clinically relevant consequences. In a clinical investigation on 50 edentulous patients receiving a single implant in the anterior mandible, neurosensory complications in the mandible were analyzed [84]. The implant position was three-dimensionally planed using a cone beam computer tomography in all cases. All patients underwent a clinical test after implant placement to reveal possible sensitivity disorders. A cotton roll was rubbed along the right and left side of the mandibular ridge, and patients were asked if they felt any difference in sensation between the two sides, or whether they noticed any kind of sensory disturbances. Thirteen patients (26%) reported transient neurosensory changes, which were all resolved after three months. According to the three-dimensional virtual implant planning, 44 patients (88%) would have had their implant touching the lingual canal.

## 4. Conclusions

Based on the presented good results with a single mandibular implant over a medium- to long-term observation period, this therapy option can be scientifically recommended for improving the retention of overdentures in the edentulous mandible of seniors. According to the authors, the main indication for this therapy option is when the use of multiple implants is not possible for financial or other reasons.

## Figures and Tables

**Table 1:** Literature overview showing first author’s name, year of publication, average age of the patients, follow-up period, characteristics of the investigation and implant survival rate.

First Author and Year	Number of Patients	Mean Age (Years)	Mean Follow-Up Period (Years)	Retention Element	Characteristics of the Investigation	Implant-Survival
Cordioli 1997 [34]	21	74.2	5	ball	Conventional loading after 4 months	100%
Krennmair 2001 [38]	9	82.2	1.5	ball	Conventional loading after 3 months	100%
Liddelow 2010 [37]	25 + 8	68.0	3	ball	Immediate loading, etched implant surfaces in 25 cases, machined implant surfaces in 8 cases	100% (etched) 62.5% (maschined)
Alsabeeha 2011 [53]	36	68.0	1	ball or Locator	Open healing with moderate loading of the healing abutment, implant loading after 6 weeks, randomized trial comparing 3 implant systems	91.7% *
Bryant 2015 [42]	42	66.6	5	ball	Open healing with moderate loading of the healing abutment, implant loading after 6 weeks, randomized trial comparing 3 implant systems	100% (94.7% for 2 implants)
Ismail 2015 [54]	10	Not specified	2	ball/magnet	Conventional loading after 4 months, randomized trial comparing ball versus magnet	100%
Tavakolizadeh 2015 [40]	10	59	1	ball	Open healing with moderate loading of the healing abutment, implant loading after 6 weeks, randomized trial comparing 1 versus 2 implants	100%
Alqutaibi 2017	28	58.2	1	Locator	Conventional loading after 3 months, randomized trial comparing 1 versus 2 implants	100%
Kronström 2017 [56]	36	53.3	5	ball	Immediate loading, randomized trial comparing 1 versus 2 implants	82.4% (81.6% for 2 implants)
Paleari 2018 [57]	11	65.0	1	ball	Conventional loading after 4 months, randomized trial comparing 1 versus 2 implants	90.9% (95% for 2 implants)
Passia 2019 [58]	11	66.7	9	ball	Conventional loading after 3 months in 5 cases, Open healing with moderate loading of the healing abutment in 6 cases, conventional loading after 3 months	100%
Asami 2020 [59]	22	74.2	1	Locator	Conventional loading after 3–5 months	95.5%
Kern 2021 [60]	158	69.3	5	ball	Immediate loading in 81 cases, conventional loading after 3 months in 77 cases	87.8% (immediate loading) 97% (conventional loading)
De Araujo 2022 [49]	11	63.5	3	ball	Conventional loading after 3 months, randomized trial comparing 1 versus 2 (removable) versus 4 (fixed) implants	100%
Passia 2022 [50]	13	at least 50 years	1	stud-att.	Conventional loading after 3 months, overdentures were successively loaded via one, two and three implants	100%
De Souza 2022 [51]	10	at least 65 years	0.5	Novaloc/Locator	Open healing, conventional loading after 8 weeks, comparison of two different attachment systems (Locator/Novaloc)	100%
Coutinho 2022 [48]	45	68.1	5	ball	Immediate loading in 38 cases, conventional loading after 3 months in 7 cases	88.9%
Ala 2022 [52]	18	65	1	stud-att.	Short implants (7 mm) placed in severely resorbed mandibles, conventional loading after 3 months	100%
De Resende 2023 [43]	23	Not specified	4	ball	Open healing, early loading after 3 weeks, randomized trial comparing 1 versus 2 implants	100% (93.7% for 2 implants)

*: all failures within one of the three implant systems used.

## Data Availability

As this is a literature overview, no new data were created.
